# Developing New Oligo Probes to Distinguish Specific Chromosomal Segments and the A, B, D Genomes of Wheat (*Triticum aestivum* L.) Using ND-FISH

**DOI:** 10.3389/fpls.2018.01104

**Published:** 2018-07-26

**Authors:** Shuyao Tang, Zongxiang Tang, Ling Qiu, Zujun Yang, Guangrong Li, Tao Lang, Wenqian Zhu, Jiehong Zhang, Shulan Fu

**Affiliations:** ^1^Province Key Laboratory of Plant Breeding and Genetics, Sichuan Agricultural University, Chengdu, China; ^2^Institute of Ecological Agriculture, Sichuan Agricultural University, Chengdu, China; ^3^Center for Informational Biology, University of Electronic Science and Technology of China, Chengdu, China

**Keywords:** wheat, chromosome, tandem repeats, ND-FISH, oligo probe

## Abstract

Non-denaturing FISH (ND-FISH) technology has been widely used to study the chromosomes of Triticeae species because of its convenience. The oligo probes for ND-FISH analysis of wheat (*Triticum aestivum* L.) chromosomes are still limited. In this study, the whole genome shotgun assembly sequences (IWGSC WGA v0.4) and the first version of the reference sequences (IWGSC RefSeq v1.0) of Chinese Spring (*T. aestivum* L.) were used to find new tandem repeats. One hundred and twenty oligo probes were designed according to the new tandem repeats and used for ND-FISH analysis of chromosomes of wheat Chinese Spring. Twenty nine of the 120 oligo probes produce clear or strong signals on wheat chromosomes. Two of the 29 oligo probes can be used to conveniently distinguish wheat A-, B-, and D-genome chromosomes. Sixteen of the 29 oligo probes only produce clear or strong signals on the subtelomeric regions of 1AS, 5AS, 7AL, 4BS, 5BS, and 3DS arms, on the telomeric regions of 1AL, 5AL, 2BS, 3BL, 6DS, and 7DL arms, on the intercalary regions of 4AL and 2DL arms, and on the pericentromeric regions of 3DL and 6DS arms. Eleven of the 29 oligo probes generate distinct signal bands on several chromosomes and they are different from those previously reported. In addition, the short and long arms of 6D chromosome have been confirmed. The new oligo probes developed in this study are useful and convenient for distinguishing wheat chromosomes or specific segments of wheat chromosomes.

## Introduction

Genomic *in situ* hybridization (GISH) and Fluorescent *in situ* hybridization (FISH) techniques are often used to identify plant chromosomes (Kato et al., 2005). GISH is extensively used to discriminate the genomes between two distant genera and within the same genus of the Triticeae tribe (Zhao et al., 2011; Fu et al., 2014; Yang et al., 2017; Grewal et al., 2017). Multicolor GISH has been successfully used to identify the A, B, and D genomes of common wheat (Mukai et al., 1993; Han et al., 2003, 2004; Zhao et al., 2011). This technology is useful for detecting the genomic constitution and chromosomal alterations of common wheat or synthetic wheat (Kato et al., 2005; Zhao et al., 2011). FISH can physically locate genes or repetitive DNA sequences on individual chromosomes of common wheat and its relatives (Danilova et al., 2012, 2017; Komuro et al., 2013). Therefore, FISH technology using genes and repetitive DNA sequences as probes is often used to establish chromosomal landmarks of the Triticeae tribe (Cuadrado and Schwarzacher, 1998; Yuan and Tomita, 2009; Wang et al., 2010; Linc et al., 2012; Cuadrado et al., 2013; Molnár et al., 2014; Danilova et al., 2017). Tandem repeats including pAs1, pSc119.2, 5S rDNA, 45S rDNA, and microsatellites such as (AAG)_n_ and (AAC)_n_ are the most commonly used probes for FISH analysis in the Triticeae species (Pedersen and Langridge, 1997; Cuadrado and Schwarzacher, 1998; Sepsi et al., 2008; Yuan and Tomita, 2009; Wang et al., 2010; Linc et al., 2012; Carvalho et al., 2013; Cuadrado et al., 2013; Molnár et al., 2014; Badaeva et al., 2015; Danilova et al., 2017). Some new tandem repeats such as pTa535, pTa713, pTa465, pTak566, pTas120, and pTas126 have been cloned from wheat (*Triticumaestivum* L.), and they are also valuable probes for wheat chromosome identification (Komuro et al., 2013). Some repetitive DNA sequences can replace the multicolor GISH to simultaneously distinguish A-, B-, and D- genome chromosomes of wheat (Komuro et al., 2013). Although the multicolor GISH using total genomic DNA as probes and the FISH using the repetitive DNA sequences as probes can unambiguously identify the different genome chromosomes or individual chromosome of wheat, their procedures are time-consuming because of the preparation of probe sequences, the labeling of DNA sequences, and the denaturing of chromosomes and probes.

Since Cuadrado et al. (2009) reported the non-denaturing FISH (ND-FISH) technology for the investigation of plant telomeres, ND-FISH assay has been widely used to study the chromosomes of Triticeae species because of its convenience (Cuadrado and Jouve, 2010; Carmona et al., 2013; Carvalho et al., 2013; Cuadrado et al., 2013; Cabo et al., 2014; Delgado et al., 2017). In these previous studies, simple sequence repeats (SSRs) were often used as probes for ND-FISH analysis. In fact, non-SSR oligonucleotide (oligo) probes can also be used for ND-FISH assays of plant chromosomes (Fu et al., 2015; Delgado et al., 2016, 2017; Li et al., 2016; Li J. et al., 2017; Du et al., 2017; Jiang et al., 2017; Kirov et al., 2017; Zhu et al., 2017). ND-FISH technology using oligo probes can also replace GISH technology to discriminate rye (*Secale cereale* L.) and *Dasypyrum villosum* chromosomes in wheat backgrounds (Fu et al., 2015; Xiao et al., 2017). Although some non-SSR oligo probes such as Oligo-pSc119.2, Oligo-pTa535, Oligo-k566, and Oligo-713 can be used for ND-FISH analysis to identify individual wheat chromosome (Fu et al., 2015; Tang et al., 2016; Zhao et al., 2016; Du et al., 2017), they are not chromosome-specific or chromosome segment-specific. Therefore, chromosome-specific or chromosome segment-specific oligo probes are needed. In this study, some tandem repeats were obtained from the genomic sequences of *T. aestivum cv.* Chinese Spring that were downloaded from the International Wheat Genome Sequencing Consortium (IWGSC) and these tandem repeats were used to design new oligo probes for ND-FISH analysis of wheat chromosomes.

## Materials and Methods

### Plant Materials

Common wheat *cv.* Chinese Spring (*T. aestivum* L.) that was kept in our laboratory was used in this study.

### Designing of New Oligo Probes

The whole genome shotgun assembly sequences (IWGSC WGA v0.4) and the first version of the reference sequences (IWGSC RefSeq v1.0) of wheat Chinese Spring were downloaded from the IWGSC^1^. Tandem Repeat Finder (TRF, version 4.09) (Benson, 1999) was used to search tandem repeats from the IWGSC WGA v0.4 and IWGSC RefSeq v1.0 (parameters: Match = 2, Mismatch = 7, Indel = 7, Probability of match = 80, Probability of indel = 10, Min score = 50, and Max period = 2000). Then, two strategies were used to filter these tandem repeats annotated by the TRF. The first, the tandem repeats were filtered using an in-house Python script in which the parameters including period size (>6), copy number (>10), and percent matches (>70). The consensus monomer sequences with identity >75% were clustered using CD-HIT (Li and Godzik, 2006) and the consensus sequences of each cluster were kept. The second, the tandem repeats annotated by the TRF were firstly divided into five classes according to the period size (6–30, 30–100, 100–200, 200–300, >300) using an in-house Python script. Each class of tandem repeats was subsequently clustered using CD-HIT (Li and Godzik, 2006) with the parameters including identity >75% and copy number >100. Then, the consensus sequences of each cluster were obtained.

Finally, the consensus sequences obtained through the two strategies were used for Nucleotide BLAST searches against the Nucleotide collection (nr/nt) database using blastn tool in National Center for Biotechnology Information. The consensus sequences that have the identity ≥70% with the published tandem repeats such as 45S rDNA, 5S rDNA, pSc119.2, pAs1, pTa-535, pTa-713, pTa-86, pTa-465, pTa-k566, pTa-s120, pTa-s126, pTa-k288, and pTa-k229 etc. were omitted, and the rest ones were used for designing oligo probes. The new oligo probes that can work are listed in **Supplementary Table S1**.

### ND-FISH Analysis

The oligo probes listed in **Supplementary Table S1** were synthesized by Tsingke Biological Technology Co. Ltd. (Beijing, China). The oligonucleotide sequences were 5′- or 3′-end-labeled with 6-carboxyfluorescein (6-FAM) or 6-carboxytetramethylrhodamine (TAMRA) (**Supplementary Table S1**). In addition, oligo probes Oligo-pSc119.2-1 and Oligo-pTa535-1 developed by Tang et al. (2014) were used to identify individual wheat chromosome. Probe Oligo-pSc119.2-1 was 5′-end-labeled with TAMRA, and probe Oligo-pTa535-1 was 5′-end-labeled with Cyanine Dye 5 (Cy5). The methods described by Han et al. (2006) were used to prepare chromosome spreads of wheat Chinese Spring. The ND-FISH analysis was performed according to the methods described by Fu et al. (2015) with some modification. That is, for the oligo probes Oligo-B and Oligo-D, when the probe mixture was dropped at the cell spreads, the room temperature should be kept at above 28°C, and the slides were immediately put in a moist box that was incubated at 42°C in advance. The high room temperature and the immediate incubating at 42°C can effectively prevent the probe Oligo-B from hybridizing with A-genome chromosomes. For new developed oligo probes, the amount of probe for each slide is listed in **Supplementary Table S1**. For each probe, three ND-FISH experiments were repeated. Images were made and processed according to the methods described by Duan et al. (2017).

### Confirmation of the Orientation of 6D Chromosome Arms

The consensus sequence of the oligo probe that only produced hybridization signal on the telomeric region of one arm of 6D chromosome was aligned using blastn tool in B2DSC^2^ against IWGSC RefSeq v1.0 of wheat Chinese Spring, and the chromosome plot was drawn using Plot tool in B2DSC.

## Results

### Obtaining the Sequences of Oligo Probes

One hundred and fourteen new tandem repeats were detected from whole genome shotgun assembly sequences (IWGSC WGA v0.4) and the first version of the reference sequences (IWGSC RefSeq v1.0) of wheat Chinese Spring. According to these tandem repeats, 120 oligo probes were designed and used for ND-FISH analysis of the root tip metaphase chromosomes of wheat Chinese Spring. Twenty-seven of the 120 oligo probes produced strong or distinct signal bands on wheat chromosomes, and two of the 120 oligo probes produced clear signals mainly on the whole B- or D-genome chromosomes. The ND-FISH signals of the 29 oligo probes are reproducible. The information of the 29 probes is listed in **Supplementary Table S1**.

### Chromosome Segment-Specific Probes

**FIGURE 1:**
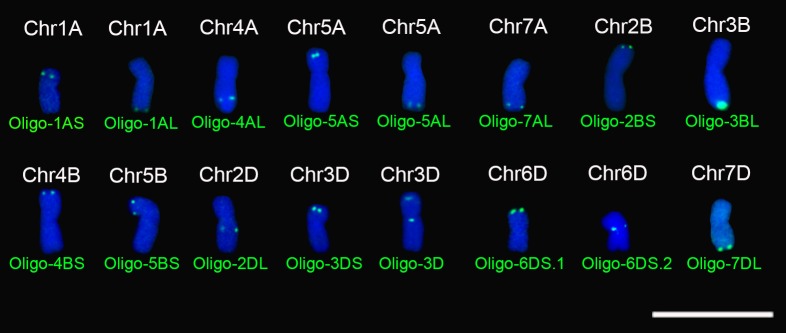
Signal patterns of the 16 oligo probes that produce signals on specific regions of metaphase chromosomes of wheat Chinese Spring. Chromosomes were counterstained with DAPI (blue). Scale bar: 30 μm.

Combined with oligo probes Oligo-pSc119.2-1 and Oligo-pTa535-1, all the new designed oligo probes were used for ND-FISH analysis of chromosomes of wheat Chinese Spring. The signal patterns of Oligo-pSc119.2-1 and Oligo-pTa535-1 can help for the recognition of individual wheat chromosome (**Supplementary Figures S1**–**S12**). Sixteen of the 29 new oligo probes only produced clear or strong signals on specific segments of wheat chromosomes (**Table 1**, **Figure 1** and **Supplementary Figures S1**–**S8**). Probes Oligo-1AS, Oligo-5AS, Oligo-7AL, Oligo-4BS, Oligo-5BS, and Oligo-3DS only generated hybridization signals on the subtelomeric regions of 1AS, 5AS, 7AL, 4BS, 5BS, and 3DS arms, respectively (**Table 1**, **Figure 1** and **Supplementary Figures S1**–**S3**). Probes Oligo-1AL, Oligo-5AL, Oligo-2BS, Oligo-3BL, Oligo-6DS.1, and Oligo-7DL only produced strong or distinct hybridization signals on the telomeric regions of 1AL, 5AL, 2BS, 3BL, 6DS, and 7DL arms, respectively (**Table 1**, **Figure 1** and **Supplementary Figures S4**–**S6**). The signals of probes Oligo-4AL and Oligo-2DL only occurred on the intercalary regions of 4AL and 2DL arms, respectively (**Table 1**, **Figure 1** and **Supplementary Figure S7**). The signal of probe Oligo-6DS.2 only appeared on the pericentromeric region of 6DS arm (**Table 1**, **Figure 1** and **Supplementary Figure S8**), and probe Oligo-3D only produced signals on the subtelomeric region of 3DS arm and the pericentromeric region of 3DL arm (**Table 1**, **Figure 1** and **Supplementary Figure S8**).

**Table 1:** Signal patterns of oligo probes developed in this study.

Probe	Nucleotide sequences	Signal location on chromosome	Probe	Nucleotide sequences	Signal location on chromosome
Oligo-1AS	5′AAAAACGCATGTCTTTAGCATTCAAAA AATGAAAAACGGTTTTTCTTGTTAAACAA3′	Subtelomeric region of 1AS	Oligo-7DL	5′CATCATTTAGGTTCTTATCGACACCG AGGCACCCTAAAAGCCTAAGCTTT3′	Telomeric region of 7DL
Oligo-1AL	5′TTCATTTTTACTCAACTCAAACTAT GGCACATACCAGGTACATCCAAA3′	Telomeric region of 1AL	Oligo-119	5′ATTTTGAGCTAGCTAAGCATATTAAGTCAT TTTGGAGCAAAAAAATGCTAAGTATAGGT3	Intercalary regions of 3BS, 6BL, and 7BS; pericentromeric region of 4BL
Oligo-4AL	5′TAACTACATGCATGAATATCCTATCAC GCCATGAAGCTAGCTAACCAACTAACC3′	Intercalary region of 4AL	Oligo-18	5′GTAGCAGTAGCAGTAGTA3′	Pericentromeric regions of 3BL and 7BL; centromeric region of 2B; intercalary regions of 1AL, 7AL, and 7BL
Oligo-5AS	5′AACTTTTTTCAAATTCGATG3′	Subtelomeric region of 5AS	Oligo-44	5′TAGCTCTACAAGCTAGTTCAAA TAATTTTACACTAGAGTTGAAC3′	Intercalary regions of 3AL, 5AL, and 5BL; telomeric region of 7AS; subtelomeric region of 7AL; pericentromeric region of 5DS
Oligo-5AL	5′ATCTATCTATAGTGATACTCCTTTGTTT GTACTAGTAATTTTGTTTGTACCCTTTC3′	Telomeric region of 5AL	Oligo-42	5′CTCGCTCGCCCAGCTGCTGCT ACTCCGGCTCTCGCTCGATCG3′	Pericentromeric regions of 1AS, 6BL, and 7BL; centromeric region of 2B; intercalary regions of 3BS, 6BS, and 7BL; centromeric and pericentromeric regions of 5B
Oligo-7AL	5′CCTTCTCCATCATTGGTGCCATGACG GAGATGTTCTTATCAATGTAAGAGACC3′	Subtelomeric region of 7AL	Oligo-45	5′CGGCCGCTCCGCGCGTCGCCATC GGTTGGTCACCTCATCACCACT3′	Pericentromeric regions of 3AS, 6AS, 4DS, 5DS, and 7DS; telomeric region of 5AL
Oligo-2BS	5′GCGTGGCCAGCCACGGCGGAACCAAC CCAATCTCGCAAAAGCGACCCCATCAC3′	Telomeric region of 2BS	Oligo-428	5′AAATGAACTGGCGAGCGGAATCGTTTGGA ACACAAAGAACCATGAAACGTGACTCTCTC3′	Pericentromeric regions of 2AS and 5DS; intercalary regions of 1DS, 1DL, 2DL, 4DL, and 6DS; subtelomeric regions of 2DS and 5DL
Oligo-3BL	5′GCCTGAAAATTACAACTTTTCGACACCGC CACGTGTAAGCCCGACCCAAACACAACCC3′	Telomeric region of 3BL	Oligo-440.1	5′CGAGAGGATCGCCGAGGGGTCGG GAGGTTGCCTAGTGTCAAAGGATTCAA3′	Subtelomeric regions of 2AS, 7AS, and 7AL
Oligo-4BS	5′GGCGGTGGGAGTGTGTTTGTCGCGCCG CAACGCCTTCTCTGTTCCTCTACGCAC3′	Subtelomeric region of 4BS	Oligo-440.2	5′CGACCCCTCGGCGATCCTCGACCCCTCGA ACCCTCGACGACCCTGGAACCCTCGACCCC3′	Intercalary regions of 3BS and 5BS; pericentromeric region of 5BL
Oligo-5BS	5′GTAGCAAGCATAGATGCTCTTCCACACGGT TTCTCTCAAGAACCGACCAATGCAGAAAC3′	Subtelomeric region of 5BS	Oligo-37	5′CTATATGATGAATTGCTA TGAGCTGCTCCTGCTGCTG3′	Pericentromeric region of 1AL; pericentromeric and intercalary regions of 7BL
Oligo-2DL	5′AAGAGAATTGCTCTGTTAA TACGAATCAATGCAACCG3′	Intercalary region of 2DL	Oligo-60	5′CCTCTCCCTCTTCTCCATTGCAAAACC ACCCCCTCTCCTCTCCATCATCTCCATT3′	Intercalary regions of 3BS and 6BS; pericentromeric regions of 5BS and 5BL
Oligo-3DS	5′CCTCGCGGGGGCGCGTCAGCGCACCCGCT GGGTGTAGCCCCCGAGATTCGGGCCGACTG3′	Subtelomeric region of 3DS	Oligo-107	5′ATTTCAGTACTGAAATACAACACCAC GATGACCCGACTAGTTCTATATCACTAA3′	Telomeric regions of 3DS, 4DS, and 4DL; intercalary region of 4AL
Oligo-3D	5′GGCGGTTGACACCATGGCGTGGAGAT GGAGATCACCACGAGGACAACCATG3′	Subtelomeric region of 3DS and pericentromeric region of 3DL	Oligo-B	5′GGTTCAGGAATAGCCT CAGGAATTGGCTCAATT3′	Mainly on the whole B-genome chromosomes
Oligo-6DS.1	5′TTTTAGCTCCCATTTAACACACC AAACTTTGACTTCCTTTTTCTTTT3′	Telomeric region of 6DS	Oligo-D	5′TACGGGTGCCAAACGAGTGTCTGAA AGACTCCTCGAGAGGAAAATGCGAA3′	Mainly on the whole D-genome chromosomes
Oligo-6DS.2	5′GAAGGGCCCCACATGTCATACTCTCACAG GGGACCATGCCCCACCTGTGTCATCCCC3′	Pericentromeric region of 6DS			

### Probes Producing Signals on Several Chromosomes

**FIGURE 2:**
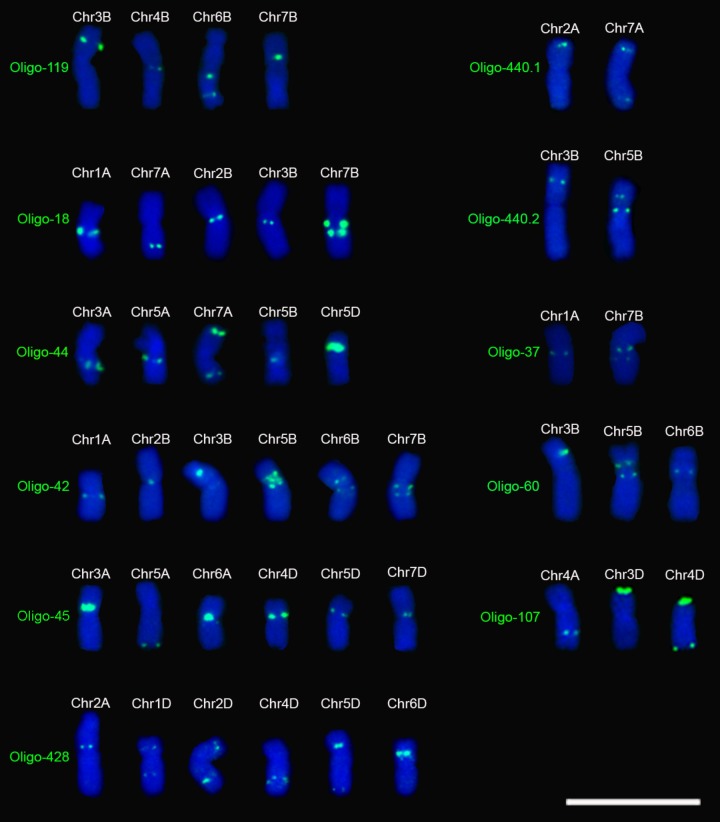
Signal patterns of the 11 oligo probes that produce signals on several metaphase chromosomes of wheat Chinese Spring. Chromosomes were counterstained with DAPI (blue). Scale bar: 30 μm.

Eleven of the 29 oligo probes generated distinct signal bands on several chromosomes and they are different from those previously reported (**Table 1**, **Figure 2** and **Supplementary Figures S9**–**S12**). All three of the probes Oligo-440.1, Oligo-440.2, and Oligo-37 produced signals on two wheat chromosomes (**Table 1**, **Figure 2** and **Supplementary Figure S9**). Both of the probes Oligo-60 and Oligo-107 generated signals on three chromosomes, and four chromosomes carried the signals of Oligo-119 (**Table 1**, **Figure 2** and **Supplementary Figure S10**). Both of the probes Oligo-18 and Oligo-44 produced signals on five chromosomes (**Table 1**, **Figure 2** and **Supplementary Figure S11**). All of the three probes Oligo-42, Oligo-45 and Oligo-428 generated signals on six chromosomes (**Table 1**, **Figure 2** and **Supplementary Figure S12**).

### B- and D-Genome Specific Probes

**FIGURE 3:**
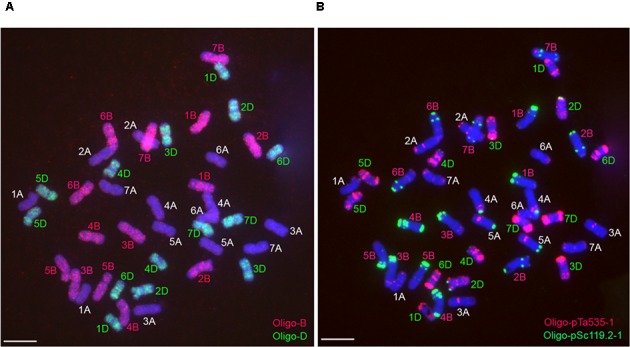
Sequential ND-FISH analysis of the metaphase chromosomes of wheat Chinese Spring. **(A)** Using probes Oligo-B (red) and Oligo-D (green) to analyze root tip metaphase chromosomes of wheat Chinese Spring. **(B)** Using probes Oligo-pTa535-1 (red) and Oligo-pSc119.2-1 (green) to analyze the same cell in **(A)** after rising the slide. Chromosomes were counterstained with DAPI (blue). Scale bar: 10 μm.

Probe Oligo-B mainly produced signals on the whole B-genome chromosomes, and probe Oligo-D mainly generated signals on the whole D-genome chromosomes (**Figure 3**). The combination of the two probes Oligo-B and Oligo-D can be used to distinguish wheat A-, B-, and D-genome chromosomes (**Figure 3**). Therefore, it is convenient to use the two oligo probes and ND-FISH analysis to identify wheat A-, B-, and D-genome chromosomes.

### The Determination of Short and Long Arms of 6D Chromosomes

**FIGURE 4:**
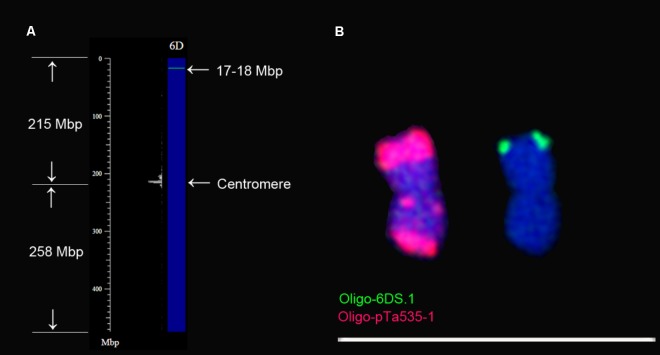
Confirmation of the orientation of 6D chromosome. **(A)** 6D chromosome plot is drawn using Plot tool in B2DSC. The green line on the chromosome plot indicates that the consensus sequence of Oligo-6DS.1 hits the sequences between the 17 and 18 Mbp regions on 6DS arm. **(B)** The location of the signal of oligo-6DS.1 is on the same arm with the one that contains one signal band of Oligo-pTa535-1. Scale bar: 70 μm.

To determine that the arm of 6D containing Oligo-6DS.1 signal is short or long arm, the consensus sequences of probe Oligo-6DS.1 was used for Nucleotide BLAST searches against the IWGSC RefSeq v1.0 of wheat Chinese Spring using blastn tool in B2DSC, and chromosome plots were drawn to view the locations of the consensus sequence on chromosomes (**Figure 4A**). The consensus sequence of Oligo-6DS.1 hits the sequences between 17 and 18 Mbp regions of 6D chromosome (**Figure 4A**). From **Figure 4A**, it can be noted that the 17–18 Mbp region should be located on the short arm of 6D chromosome (6DS). Therefore, the signal of probe Oligo-6DS.1 should be located on the telomeric region of 6DS arm (**Figures 4A,B**). The 6D chromosome can be identified using probe Oligo-pTa535-1 (**Figure 4B**). Probe Oligo-pTa535-1 produces a very strong signal band on one arm of 6D chromosome, and two clear signal bands on the other arm (**Figure 4B**). According to the **Figures 4A,B**, therefore, the arm carrying one signal band of Oligo-pTa535-1 should be the 6DS arm.

## Discussion

### Developing New Oligo Probes for ND-FISH Analysis of Common Wheat Chromosomes

ND-FISH technology has provided a convenient and efficient way to identify the chromosomes of Triticeae species (Cuadrado and Jouve, 2010; Carmona et al., 2013; Carvalho et al., 2013; Cuadrado et al., 2013; Cabo et al., 2014; Tang et al., 2014; Fu et al., 2015; Delgado et al., 2016, 2017; Li et al., 2016; Li J. et al., 2017; Li H. et al., 2017; Du et al., 2017; Duan et al., 2017; Jiang et al., 2017; Xiao et al., 2017). The key step for a successful ND-FISH analysis is to obtaining suitable oligo probes. Most of the previously reported oligo probes that produce signals on wheat chromosomes were developed from tandem repeats. It is easy to obtain SSR oligo probes. For developing new non-SSR oligo probes, the new repetitive DNA sequences must be obtained firstly. It has been reported that DNase-I-digested genomic TA cloning procedure is highly effective to get FISH-positive repeated sequences of barley (*Hordeum vulgare* L.) (Kato, 2011). This procedure is equally valid for common wheat (Komuro et al., 2013). With the development of next generation sequencing technology, more and more genomes of species have been successfully sequenced, and this provides a convenient way to search new FISH-positive repeated sequences. Using the next generation sequencing data of *Allium fistulosum*, two FISH-positive tandem repeats HAT58 and CAT36 were detected (Kirov et al., 2017). Based on the reference genome sequences of cucumber (*Cucumis sativus*), a bioinformatic pipeline was used to develop bulked oligo probes, in which repetitive DNA sequences were omitted (Han et al., 2015). The bulked oligo probes can be used to identify single cucumber chromosome and to paint homeologous chromosomes in other *Cucumis* species (Han et al., 2015). The same way was used to develop oligo probes that can build a bar code or banding pattern to uniquely label each of the 12 chromosomes of potato (Braz et al., 2018). The released whole genome shotgun assembly sequences (IWGSC WGA v0.4) and the first version of the reference sequences (IWGSC RefSeq v1.0) of wheat Chinese Spring provide a good resource for searching new tandem repeats of common wheat and designing new ND-FISH-positive oligo probes. In the present study, 29 ND-FISH-positive oligo probes are obtained from the reference genome sequence of wheat Chinese Spring. Another excellent method, graph-based approach, was also used to search repetitive sequences from next-generation sequencing data (Novák et al., 2010). In this method, two-step analysis including partitioning the data into clusters of overlapping reads representing individual repeated elements and further characterization of these clusters was used to find repetitive elements including tandem and dispersed repeats (Novák et al., 2010). The graph-based approach mainly focuses on distinguishing basic types of repeats by graph representation and investigating sequence variability within repeat families (Novák et al., 2010). In this study, tandem repeats were focused on and the software Tandem Repeats Finder (Benson, 1999) was used to search tandem repeats from wheat genomic sequences. Then all tandem repeats are clustered into clusters by CD-HIT program that meet 75% similarity threshold, and the consensus sequence of each cluster that has not been reported was selected to design oligonucleotide probe. Therefore, in this study, the dispersed repeats were excluded firstly and the tandem repeats that are suitable for designing oligo probes can be effectively found.

### The Applications of the New Oligo Probes Developed in This Study

Simple sequence repeat and non-SSR oligo probes such as (AAG)_n_, (AAC)_n_, Oligo-pSc119.2, Oligo-pTa535, Oligo-pTa71, Oligo-s120, Oligo-275, Oligo-k566, and Oligo-713 have already been used to identify individual wheat chromosome (Cuadrado and Schwarzacher, 1998; Cuadrado et al., 2000; Carvalho et al., 2013; Mirzaghaderi et al., 2014; Tang et al., 2014, 2016; Fu et al., 2015; Zhang et al., 2015; Li et al., 2016; Li J. et al., 2017; Li H. et al., 2017; Yang et al., 2016; Zhao et al., 2016, 2018; Delgado et al., 2017; Du et al., 2017; Duan et al., 2017; Jiang et al., 2017; Xiao et al., 2017). However, these oligo probes produce their signals on several wheat chromosomes and they are not chromosome-specific or chromosome segment-specific. Generally, using these oligo probes, wheat chromosomes can be well distinguished only when they are intact. Some segments of wheat chromosomes are difficult to be identified if they are broken off from their original chromosomes because of the lack of chromosome-specific or chromosome segment-specific probes. Bulked oligo probes that are non-repetitive DNA sequences can be used to identify a single entire chromosome and special segment of a chromosome (Han et al., 2015). In the present study, 16 oligo probes that were derived from tandem repeats are chromosome segment-specific and they can also be used to recognize special segments of some wheat chromosomes. In addition, a method named fluorescent *in situ* hybridization in suspension (FISHIS) has been developed to effectively isolate individual chromosome of wheat and its relatives (Giorgi et al., 2013; Lucretti et al., 2014). In this method, synthetic and fluorescence-labeled SSR oligo probes were used, and heat denaturation of chromosomes was avoided (Giorgi et al., 2013; Lucretti et al., 2014). Therefore, the 16 oligo probes developed in this study might be helpful for the flow sorting and isolation of individual wheat chromosome using FISHIS technology because these oligo probes are chromosome segment-specific and they can be used for ND-FISH analysis.

It has already been reported that chromosomal rearrangements are ubiquitous among polyploidy wheat accessions (Badaeva et al., 2007). Multicolor GISH technology can be used to effectively detect the reciprocally translocated chromosomes involving the A, B, and D genome of wheat (Mukai et al., 1993; Han et al., 2004; Kato et al., 2005). In this study, the combination of the oligo probes Oligo-B and Oligo-D can easily distinguish the A, B, and D genomes of common wheat through ND-FISH analysis. Therefore, the two oligo probes can replace the multicolor GISH to fastly identify some reciprocal translocations among the three genomes of common wheat.

For the other new oligo probes in this study, they produced the FISH signal patterns that are different from those previously reported (Pedersen and Langridge, 1997; Sepsi et al., 2008; Komuro et al., 2013; Tang et al., 2014; Du et al., 2017). The FISH signal patterns built in the present study provide one more new chromosomal landmarks of common wheat.

### The Orientation of 6D Chromosome

Tandem repeats pAs1 mainly produce signal bands on D-genome chromosomes, and the arm of 6D chromosome that contains one signal band of pAs1 was regarded as the long arm (Mukai et al., 1993; Pedersen and Langridge, 1997). Tandem repeats pTa-535 produce the same signal pattern as the one produced by pAs1 on 6D chromosome (Komuro et al., 2013). Again, the arm that contains one signal band of pTa-535 was regarded as the 6DL arm (Komuro et al., 2013). Oligo probe Oligo-pTa535 can replace the role of pTa-535 to identify wheat chromosomes (Tang et al., 2014). The arm of 6D chromosome that carries one signal band of Oligo-pTa535 was regarded as 6DS arm (Tang et al., 2014). In the present study, the reference sequences of wheat Chinese Spring (IWGSC RefSeq v1.0) combined with ND-FISH analysis and blastn tool in B2DSC was used to confirm the orientation of 6D chromosome, and the results indicate that the arm carrying one signal band of Oligo-pTa535-1 should be the 6DS arm. That is, the arm of 6D chromosome that contains one signal band of pAs1 or pTa-535 should be 6DS arm. B2DSC was established by Dr. Zujun Yang, Center for Informational Biology, University of Electronic Science and Technology of China. It provides a view of the distribution of the homologous sequences of oligonucleotide probes on chromosomes, mainly for wheat research. Based on the newly released IWGSC RefSeq v1.0 of wheat Chinese Spring, the general distribution patterns of repeated sequences in the genome can be viewed through drawing a chromosome plot.

## Conclusion

The whole genome shotgun assembly sequences (IWGSC WGA v0.4) and the first version of the reference sequences (IWGSC RefSeq v1.0) of wheat Chinese Spring were used to develop new oligo probes for ND-FISH analysis of wheat chromosomes. Two oligo probes can be used to identify wheat A-, B-, and D-genome chromosomes, 16 oligo probes can be used to identify specific segments of wheat chromosomes, and 11 oligo probes give new chromosomal landmarks of common wheat. These oligo probes are useful and convenient for distinguishing wheat chromosomes or specific segments of wheat chromosomes. In addition, the short and long arms of 6D chromosome have been confirmed.

## Author Contributions

SF and ZT designed the study, analyzed the data, and wrote the manuscript. SF, ZT, ST, TL, and ZY analyzed the genomic sequences of wheat Chinese Spring and designed the oligo probes. LQ, GL, WZ, and JZ performed the experiments.

## Conflict of Interest Statement

The authors declare that the research was conducted in the absence of any commercial or financial relationships that could be construed as a potential conflict of interest.

## Footnotes

1 http://www.wheatgenome.org

2 http://mcgb.uestc.edu.cn/b2dsc

## Supplementary Material

FIGURE S1 ND-FISH analysis of root tip metaphase chromosomes of wheat Chinese Spring. **(A,B)** ND-FISH analysis of the same cell using Oligo-pTa535-1 (red), Oligo-pSc119.2-1 (green), Oligo-1AS (green) as probes. **(C,D)** ND-FISH analysis of the same cell using Oligo-pTa535-1 (red), Oligo-pSc119.2-1 (green), Oligo-5AS (green) as probes. Chromosomes were counterstained with DAPI (blue). Scale bar: 10 μm.

FIGURE S2 ND-FISH analysis of root tip metaphase chromosomes of wheat Chinese Spring. **(A,B)** ND-FISH analysis of the same cell using Oligo-pTa535-1 (red), Oligo-pSc119.2-1 (green), Oligo-7AL (green) as probes. **(C,D)** ND-FISH analysis of the same cell using Oligo-pTa535-1 (red), Oligo-pSc119.2-1 (green), Oligo-4BS (green) as probes. Chromosomes were counterstained with DAPI (blue). Scale bar: 10 μm.

FIGURE S3 ND-FISH analysis of root tip metaphase chromosomes of wheat Chinese Spring. **(A,B)** ND-FISH analysis of the same cell using Oligo-pTa535-1 (red), Oligo-pSc119.2-1 (green), Oligo-5BS (green) as probes. **(C,D)** ND-FISH analysis of the same cell using Oligo-pTa535-1 (red), Oligo-pSc119.2-1 (green), Oligo-3DS (green) as probes. Chromosomes were counterstained with DAPI (blue). Scale bar: 10 μm.

FIGURE S4 ND-FISH analysis of root tip metaphase chromosomes of wheat Chinese Spring. **(A,B)** ND-FISH analysis of the same cell using Oligo-pTa535-1 (red), Oligo-pSc119.2-1 (green), Oligo-1AL (green) as probes. **(C,D)** ND-FISH analysis of the same cell using Oligo-pTa535-1 (red), Oligo-pSc119.2-1 (green), Oligo-5AL (green) as probes. Chromosomes were counterstained with DAPI (blue). Scale bar: 10 μm.

FIGURE S5 ND-FISH analysis of root tip metaphase chromosomes of wheat Chinese Spring. **(A,B)** ND-FISH analysis of the same cell using Oligo-pTa535-1 (red), Oligo-pSc119.2-1 (green), Oligo-2BS (green) as probes. **(C,D)** ND-FISH analysis of the same cell using Oligo-pTa535-1 (red), Oligo-pSc119.2-1 (green), Oligo-3BL (green) as probes. Chromosomes were counterstained with DAPI (blue). Scale bar: 10 μm.

FIGURE S6 ND-FISH analysis of root tip metaphase chromosomes of wheat Chinese Spring. **(A,B)** ND-FISH analysis of the same cell using Oligo-pTa535-1 (red), Oligo-pSc119.2-1 (green), Oligo-6DS.1 (green) as probes. **(C,D)** ND-FISH analysis of the same cell using Oligo-pTa535-1 (red), Oligo-pSc119.2-1 (green), Oligo-7DL (green) as probes. Chromosomes were counterstained with DAPI (blue). Scale bar: 10 μm.

FIGURE S7 ND-FISH analysis of root tip metaphase chromosomes of wheat Chinese Spring. **(A,B)** ND-FISH analysis of the same cell using Oligo-pTa535-1 (red), Oligo-pSc119.2-1 (green), Oligo-4AL (green) as probes. **(C,D)** ND-FISH analysis of the same cell using Oligo-pTa535-1 (red), Oligo-pSc119.2-1 (green), Oligo-2DL (green) as probes. Chromosomes were counterstained with DAPI (blue). Scale bar: 10 μm.

FIGURE S8 ND-FISH analysis of root tip metaphase chromosomes of wheat Chinese Spring. **(A,B)** ND-FISH analysis of the same cell using Oligo-pTa535-1 (red), Oligo-pSc119.2-1 (green), Oligo-3D (green) as probes. **(C,D)** ND-FISH analysis of the same cell using Oligo-pTa535-1 (red), Oligo-pSc119.2-1 (green), Oligo-6DS.2 (green) as probes. Chromosomes were counterstained with DAPI (blue). Scale bar: 10 μm.

FIGURE S9 ND-FISH analysis of root tip metaphase chromosomes of wheat Chinese Spring. **(A,B)** ND-FISH analysis of the same cell using Oligo-pTa535-1 (red), Oligo-pSc119.2-1 (green), Oligo-440.1 (green) as probes. **(C,D)** ND-FISH analysis of the same cell using Oligo-pTa535-1 (red), Oligo-pSc119.2-1 (green), Oligo-440.2 (green) as probes. **(E,F)** ND-FISH analysis of the same cell using Oligo-pTa535-1 (red), Oligo-pSc119.2-1 (green), Oligo-37 (green) as probes. Chromosomes were counterstained with DAPI (blue). Scale bar: 10 μm.

FIGURE S10 ND-FISH analysis of root tip metaphase chromosomes of what Chinese Spring. **(A,B)** ND-FISH analysis of the same cell using Oligo-pTa535-1 (red), Oligo-pSc119.2-1 (green), Oligo-60 (green) as probes. **(C,D)** ND-FISH analysis of the same cell using Oligo-pTa535-1 (red), Oligo-pSc119.2-1 (green), Oligo-107 (green) as probes. **(E,F)** ND-FISH analysis of the same cell using Oligo-pTa535-1 (red), Oligo-pSc119.2-1 (green), Oligo-119 (green) as probes. Chromosomes were counterstained with DAPI (blue). Scale bar: 10 μm.

FIGURE S11 ND-FISH analysis of root tip metaphase chromosomes of wheat Chinese Spring. **(A,B)** ND-FISH analysis of the same cell using Oligo-pTa535-1 (red), Oligo-pSc119.2-1 (green), Oligo-18 (green) as probes. **(C,D)** ND-FISH analysis of the same cell using Oligo-pTa535-1 (red), Oligo-pSc119.2-1 (green), Oligo-44 (green) as probes. Chromosomes were counterstained with DAPI (blue). Scale bar: 10 μm.

FIGURE S12 ND-FISH analysis of root tip metaphase chromosomes of wheat Chinese Spring. **(A,B)** ND-FISH analysis of the same cell using Oligo-pTa535-1 (red), Oligo-pSc119.2-1 (green), Oligo-42 (green) as probes. **(C,D)** ND-FISH analysis of the same cell using Oligo-pTa535-1 (red), Oligo-pSc119.2-1 (green), Oligo-45 (green) as probes. **(E,F)** ND-FISH analysis of the same cell using Oligo-pTa535-1 (red), Oligo-pSc119.2-1 (green), Oligo-448 (green) as probes. Chromosomes were counterstained with DAPI (blue). Scale bar: 10 μm.

TABLE S1 Information of the new oligo probes.
