# Supplementary figures and images for: Developing New Oligo Probes to Distinguish Specific Chromosomal Segments and the A, B, D Genomes of Wheat (Triticum aestivum L.) Using ND-FISH
Source: Front Plant Sci. 2018 Jul 26;9:1104. doi: 10.3389/fpls.2018.01104 (PMC6070686; doi:10.3389/fpls.2018.01104)

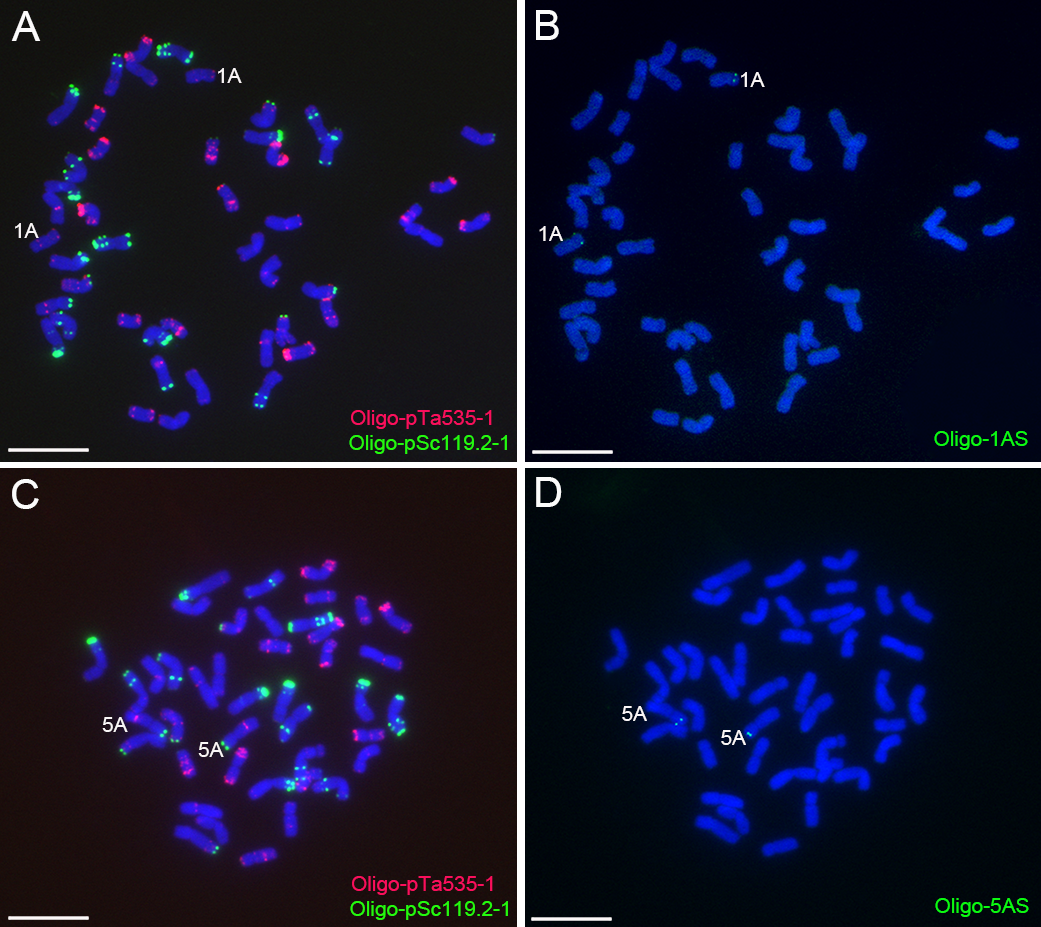

Supplement: FIGURE S1 — ND-FISH analysis of root tip metaphase chromosomes of wheat Chinese Spring. (A,B) ND-FISH analysis of the same cell using Oligo-pTa535-1 (red), Oligo-pSc119.2-1 (green), Oligo-1AS (green) as probes. (C,D) ND-FISH analysis of the same cell using Oligo-pTa535-1 (red), Oligo-pSc119.2-1 (green), Oligo-5AS (green) as probes. Chromosomes were counterstained with DAPI (blue). Scale bar: 10 μm. [file Image_1.TIF]

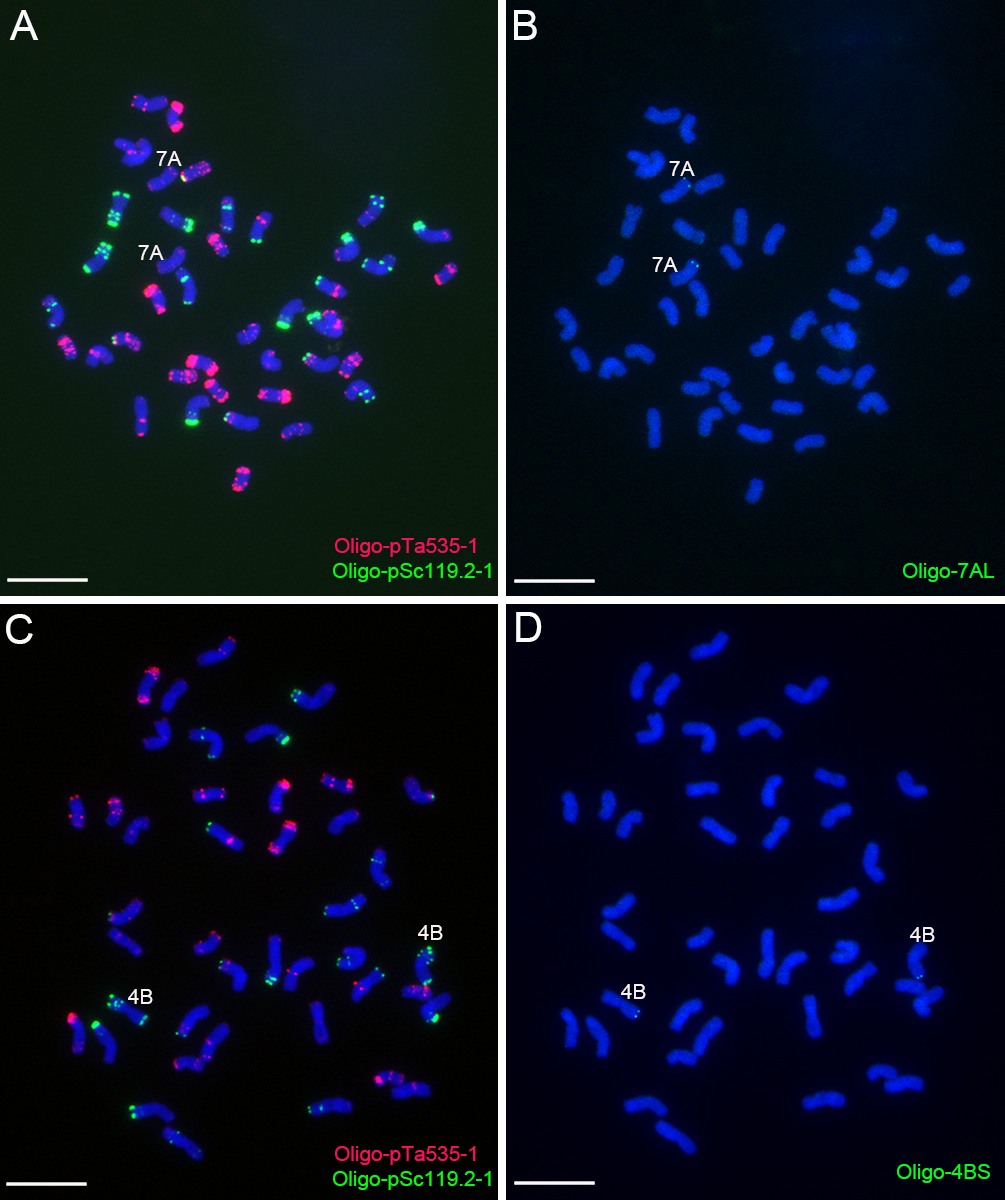

Supplement: FIGURE S2 — ND-FISH analysis of root tip metaphase chromosomes of wheat Chinese Spring. (A,B) ND-FISH analysis of the same cell using Oligo-pTa535-1 (red), Oligo-pSc119.2-1 (green), Oligo-7AL (green) as probes. (C,D) ND-FISH analysis of the same cell using Oligo-pTa535-1 (red), Oligo-pSc119.2-1 (green), Oligo-4BS (green) as probes. Chromosomes were counterstained with DAPI (blue). Scale bar: 10 μm. [file Image_2.TIF]

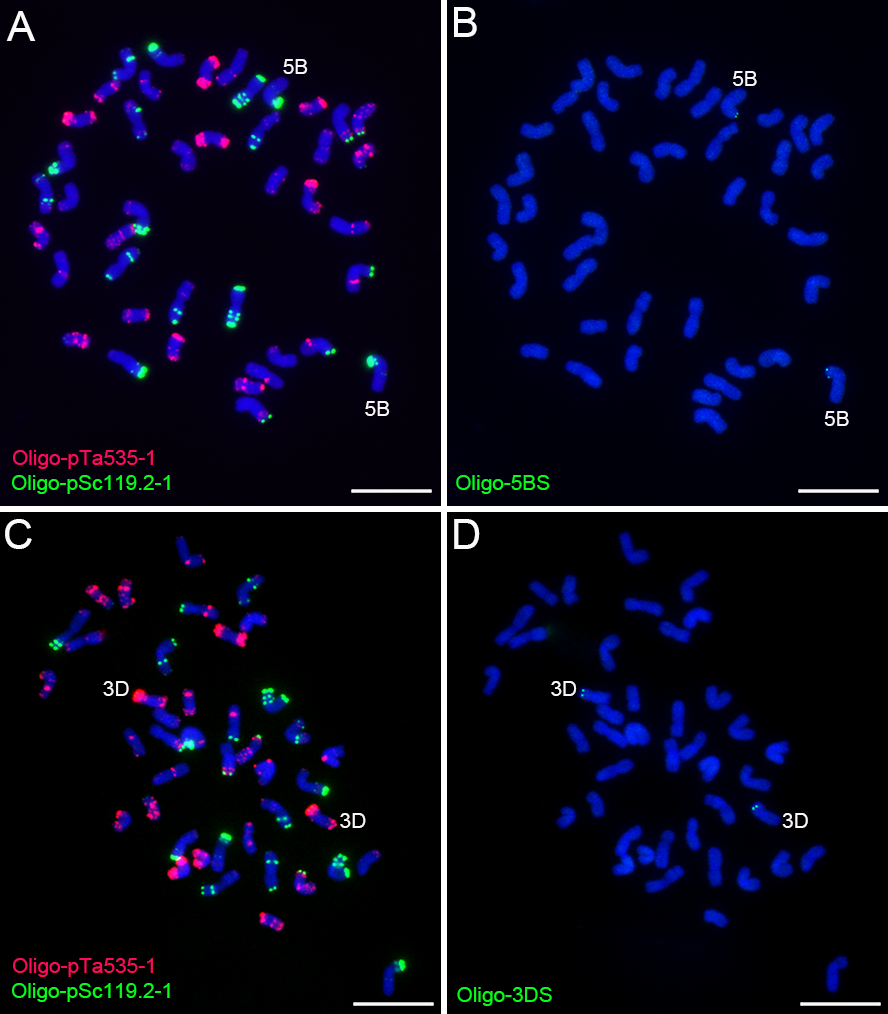

Supplement: FIGURE S3 — ND-FISH analysis of root tip metaphase chromosomes of wheat Chinese Spring. (A,B) ND-FISH analysis of the same cell using Oligo-pTa535-1 (red), Oligo-pSc119.2-1 (green), Oligo-5BS (green) as probes. (C,D) ND-FISH analysis of the same cell using Oligo-pTa535-1 (red), Oligo-pSc119.2-1 (green), Oligo-3DS (green) as probes. Chromosomes were counterstained with DAPI (blue). Scale bar: 10 μm. [file Image_3.TIF]

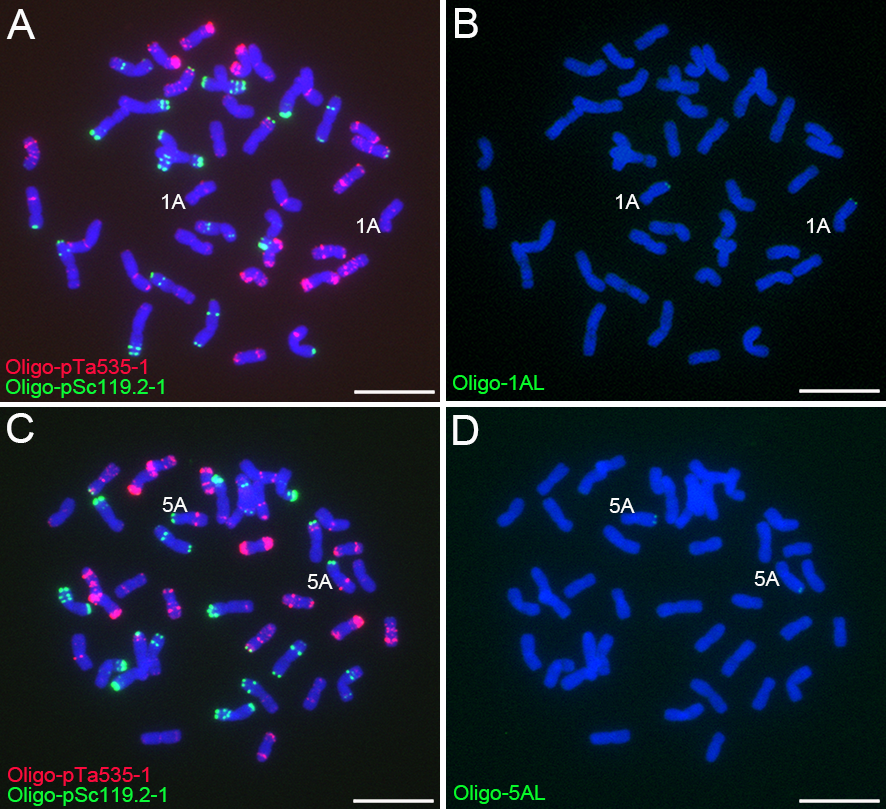

Supplement: FIGURE S4 — ND-FISH analysis of root tip metaphase chromosomes of wheat Chinese Spring. (A,B) ND-FISH analysis of the same cell using Oligo-pTa535-1 (red), Oligo-pSc119.2-1 (green), Oligo-1AL (green) as probes. (C,D) ND-FISH analysis of the same cell using Oligo-pTa535-1 (red), Oligo-pSc119.2-1 (green), Oligo-5AL (green) as probes. Chromosomes were counterstained with DAPI (blue). Scale bar: 10 μm. [file Image_4.TIF]

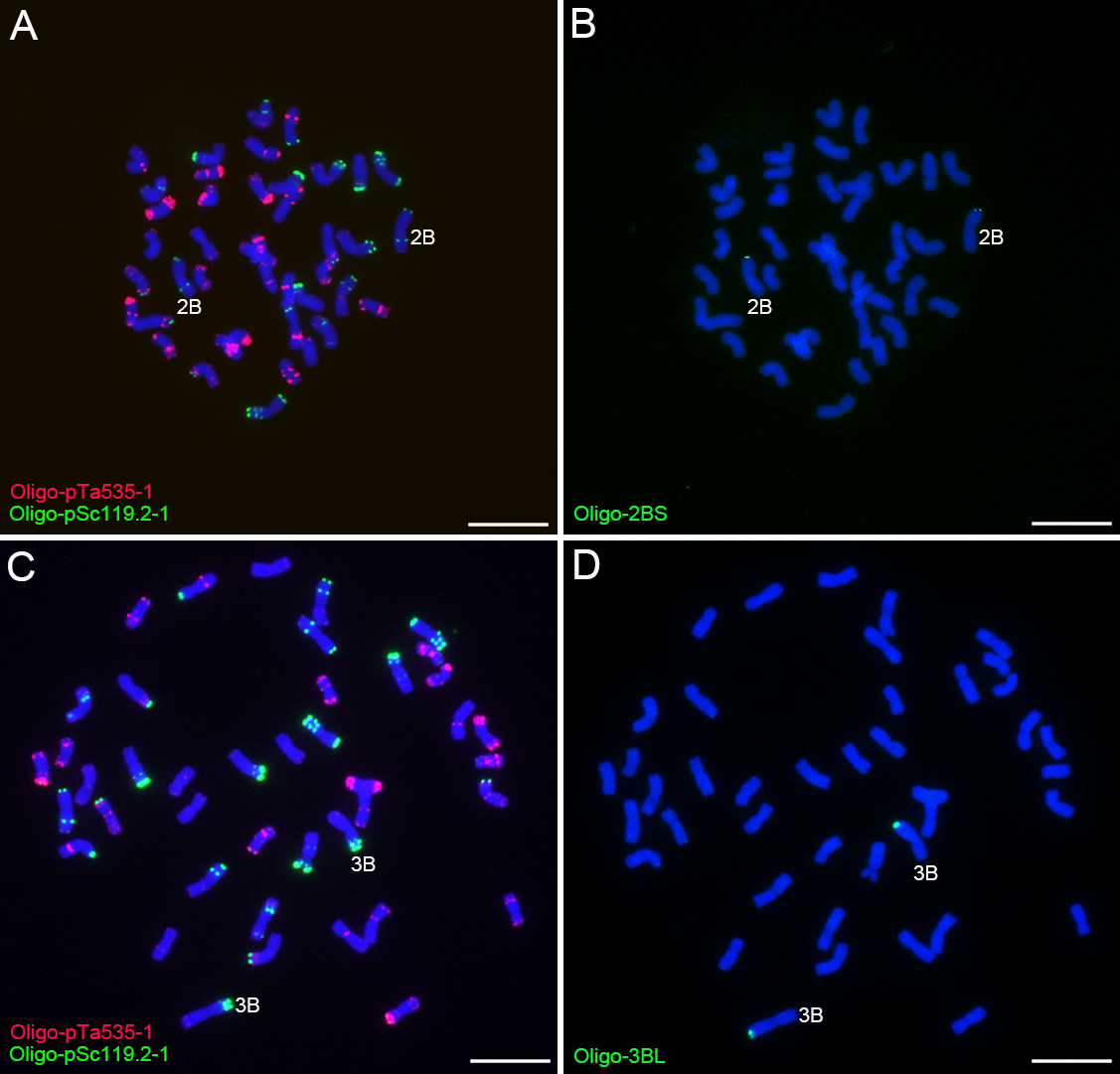

Supplement: FIGURE S5 — ND-FISH analysis of root tip metaphase chromosomes of wheat Chinese Spring. (A,B) ND-FISH analysis of the same cell using Oligo-pTa535-1 (red), Oligo-pSc119.2-1 (green), Oligo-2BS (green) as probes. (C,D) ND-FISH analysis of the same cell using Oligo-pTa535-1 (red), Oligo-pSc119.2-1 (green), Oligo-3BL (green) as probes. Chromosomes were counterstained with DAPI (blue). Scale bar: 10 μm. [file Image_5.TIF]

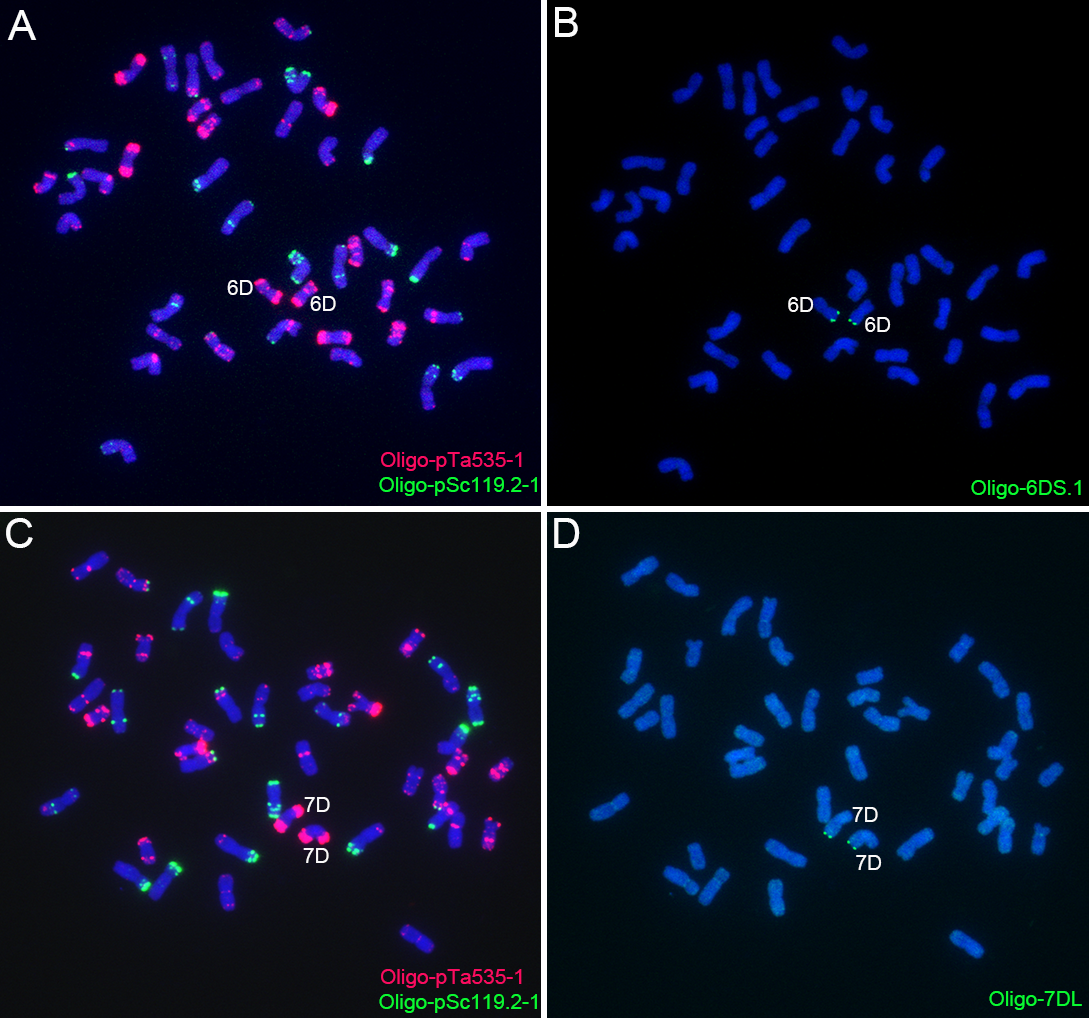

Supplement: FIGURE S6 — ND-FISH analysis of root tip metaphase chromosomes of wheat Chinese Spring. (A,B) ND-FISH analysis of the same cell using Oligo-pTa535-1 (red), Oligo-pSc119.2-1 (green), Oligo-6DS.1 (green) as probes. (C,D) ND-FISH analysis of the same cell using Oligo-pTa535-1 (red), Oligo-pSc119.2-1 (green), Oligo-7DL (green) as probes. Chromosomes were counterstained with DAPI (blue). Scale bar: 10 μm. [file Image_6.TIF]

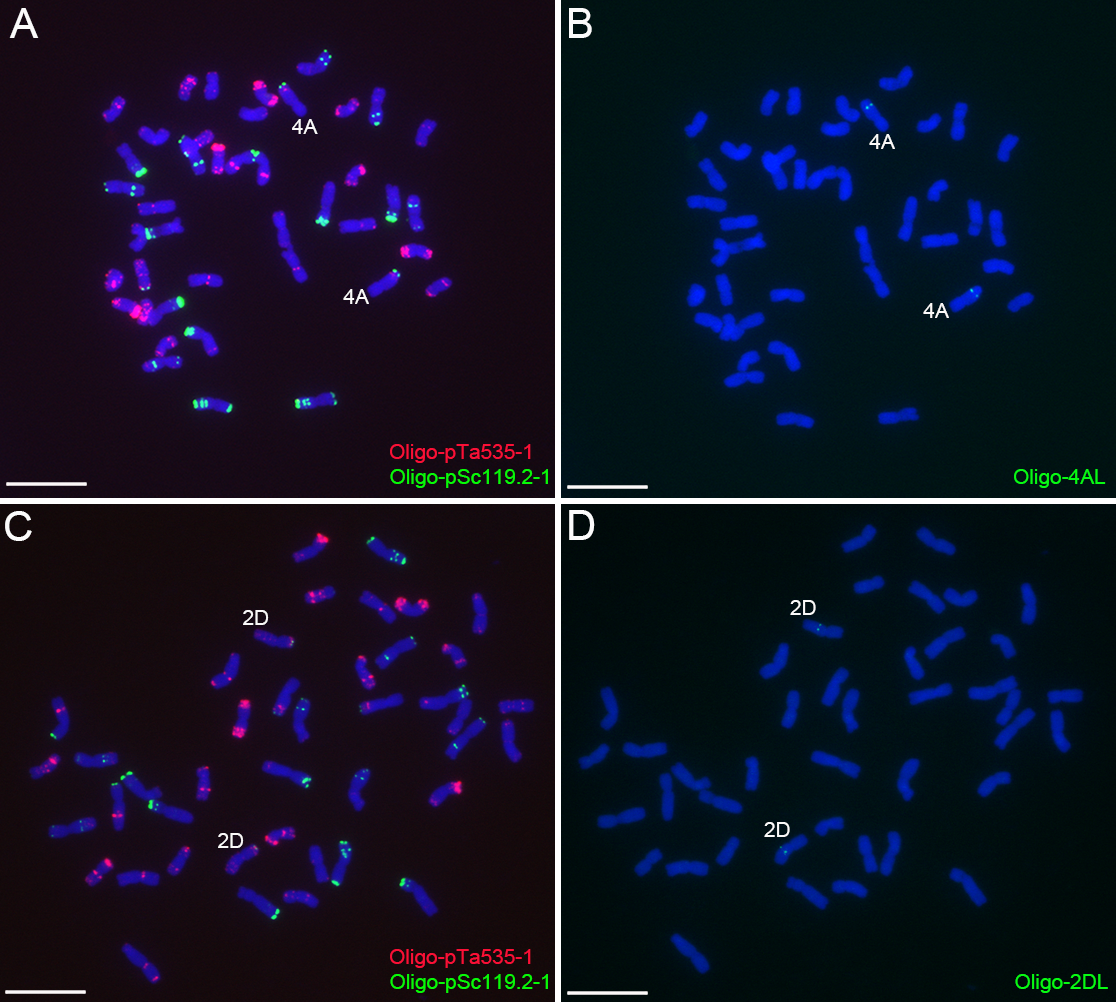

Supplement: FIGURE S7 — ND-FISH analysis of root tip metaphase chromosomes of wheat Chinese Spring. (A,B) ND-FISH analysis of the same cell using Oligo-pTa535-1 (red), Oligo-pSc119.2-1 (green), Oligo-4AL (green) as probes. (C,D) ND-FISH analysis of the same cell using Oligo-pTa535-1 (red), Oligo-pSc119.2-1 (green), Oligo-2DL (green) as probes. Chromosomes were counterstained with DAPI (blue). Scale bar: 10 μm. [file Image_7.TIF]

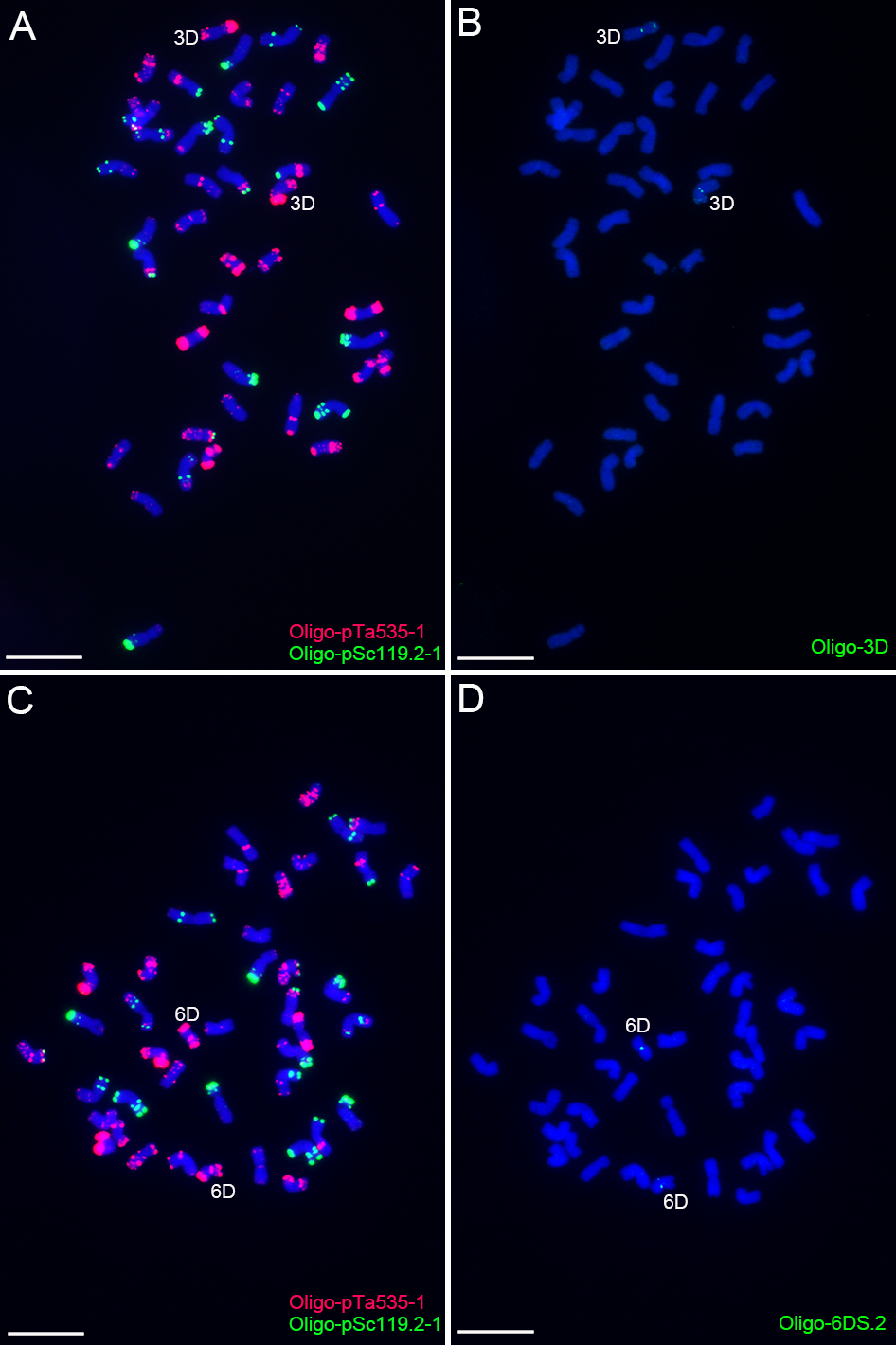

Supplement: FIGURE S8 — ND-FISH analysis of root tip metaphase chromosomes of wheat Chinese Spring. (A,B) ND-FISH analysis of the same cell using Oligo-pTa535-1 (red), Oligo-pSc119.2-1 (green), Oligo-3D (green) as probes. (C,D) ND-FISH analysis of the same cell using Oligo-pTa535-1 (red), Oligo-pSc119.2-1 (green), Oligo-6DS.2 (green) as probes. Chromosomes were counterstained with DAPI (blue). Scale bar: 10 μm. [file Image_8.TIF]

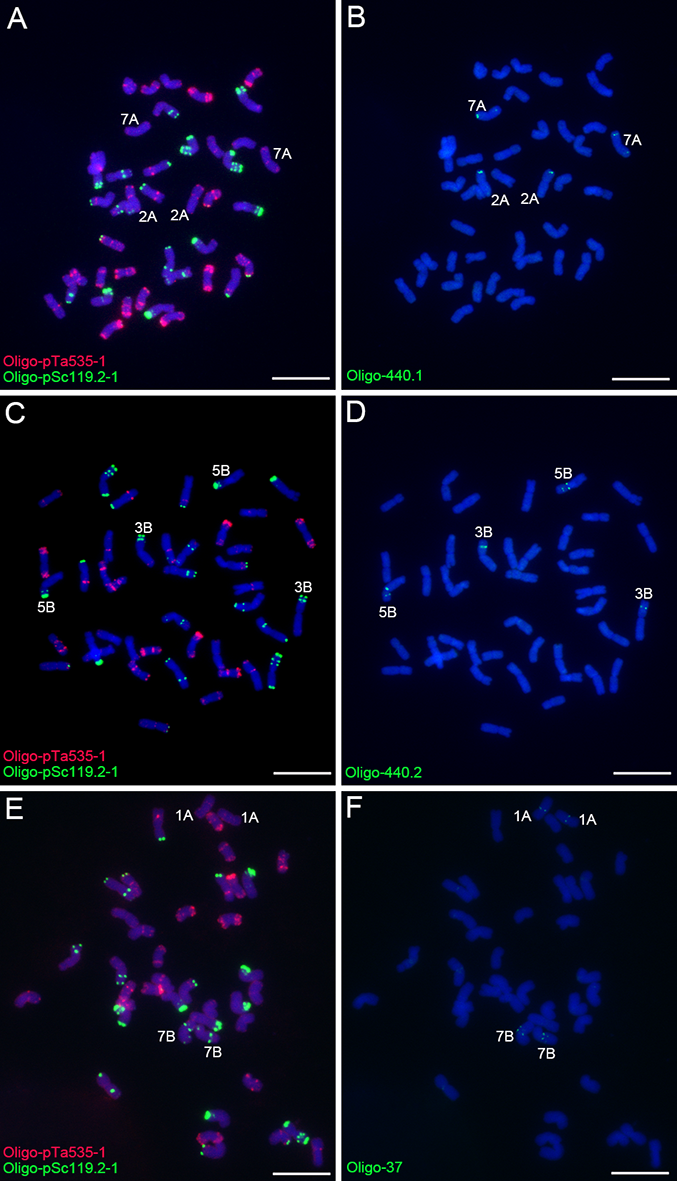

Supplement: FIGURE S9 — ND-FISH analysis of root tip metaphase chromosomes of wheat Chinese Spring. (A,B) ND-FISH analysis of the same cell using Oligo-pTa535-1 (red), Oligo-pSc119.2-1 (green), Oligo-440.1 (green) as probes. (C,D) ND-FISH analysis of the same cell using Oligo-pTa535-1 (red), Oligo-pSc119.2-1 (green), Oligo-440.2 (green) as probes. (E,F) ND-FISH analysis of the same cell using Oligo-pTa535-1 (red), Oligo-pSc119.2-1 (green), Oligo-37 (green) as probes. Chromosomes were counterstained with DAPI (blue). Scale bar: 10 μm. [file Image_9.TIF]

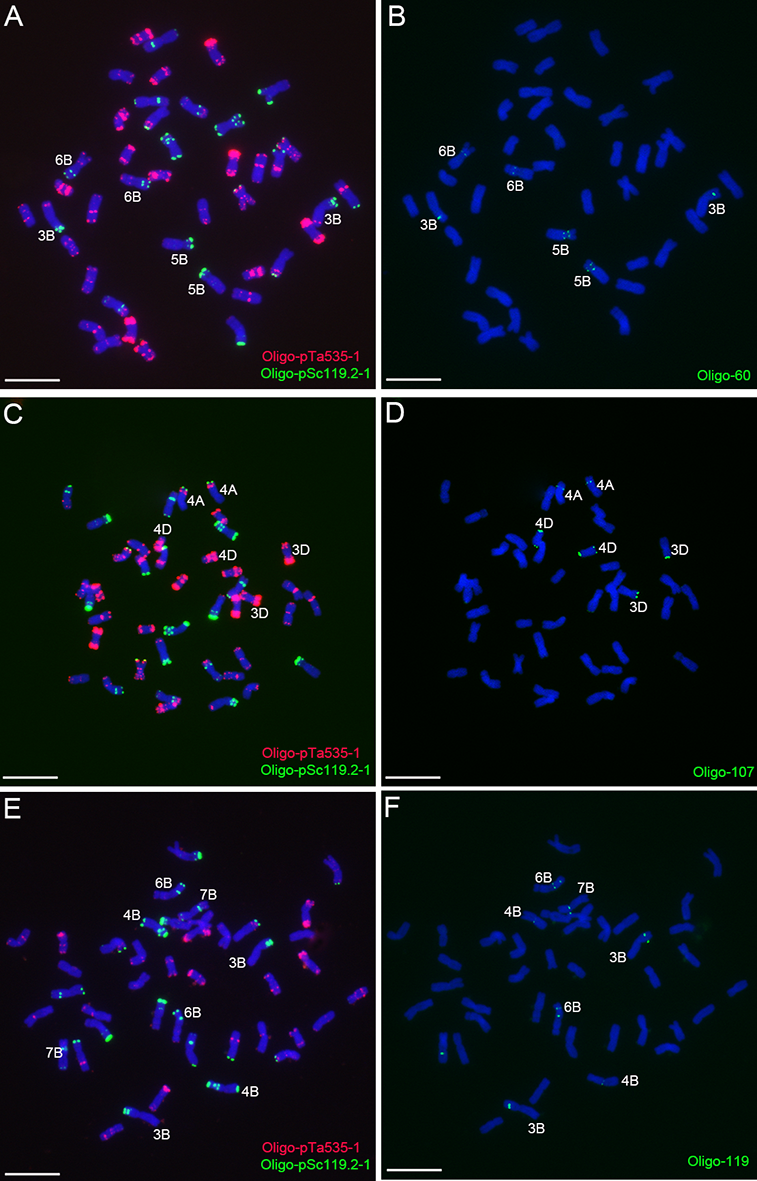

Supplement: FIGURE S10 — ND-FISH analysis of root tip metaphase chromosomes of what Chinese Spring. (A,B) ND-FISH analysis of the same cell using Oligo-pTa535-1 (red), Oligo-pSc119.2-1 (green), Oligo-60 (green) as probes. (C,D) ND-FISH analysis of the same cell using Oligo-pTa535-1 (red), Oligo-pSc119.2-1 (green), Oligo-107 (green) as probes. (E,F) ND-FISH analysis of the same cell using Oligo-pTa535-1 (red), Oligo-pSc119.2-1 (green), Oligo-119 (green) as probes. Chromosomes were counterstained with DAPI (blue). Scale bar: 10 μm. [file Image_10.TIF]

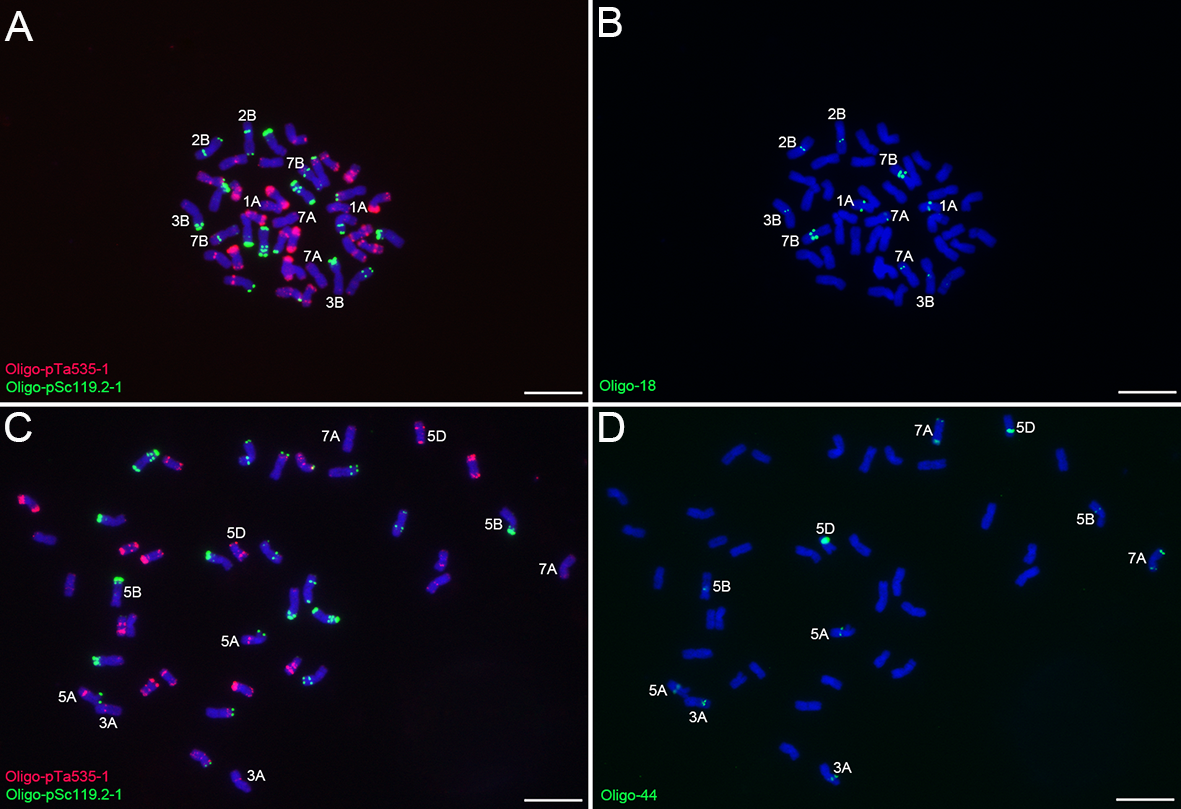

Supplement: FIGURE S11 — ND-FISH analysis of root tip metaphase chromosomes of wheat Chinese Spring. (A,B) ND-FISH analysis of the same cell using Oligo-pTa535-1 (red), Oligo-pSc119.2-1 (green), Oligo-18 (green) as probes. (C,D) ND-FISH analysis of the same cell using Oligo-pTa535-1 (red), Oligo-pSc119.2-1 (green), Oligo-44 (green) as probes. Chromosomes were counterstained with DAPI (blue). Scale bar: 10 μm. [file Image_11.TIF]

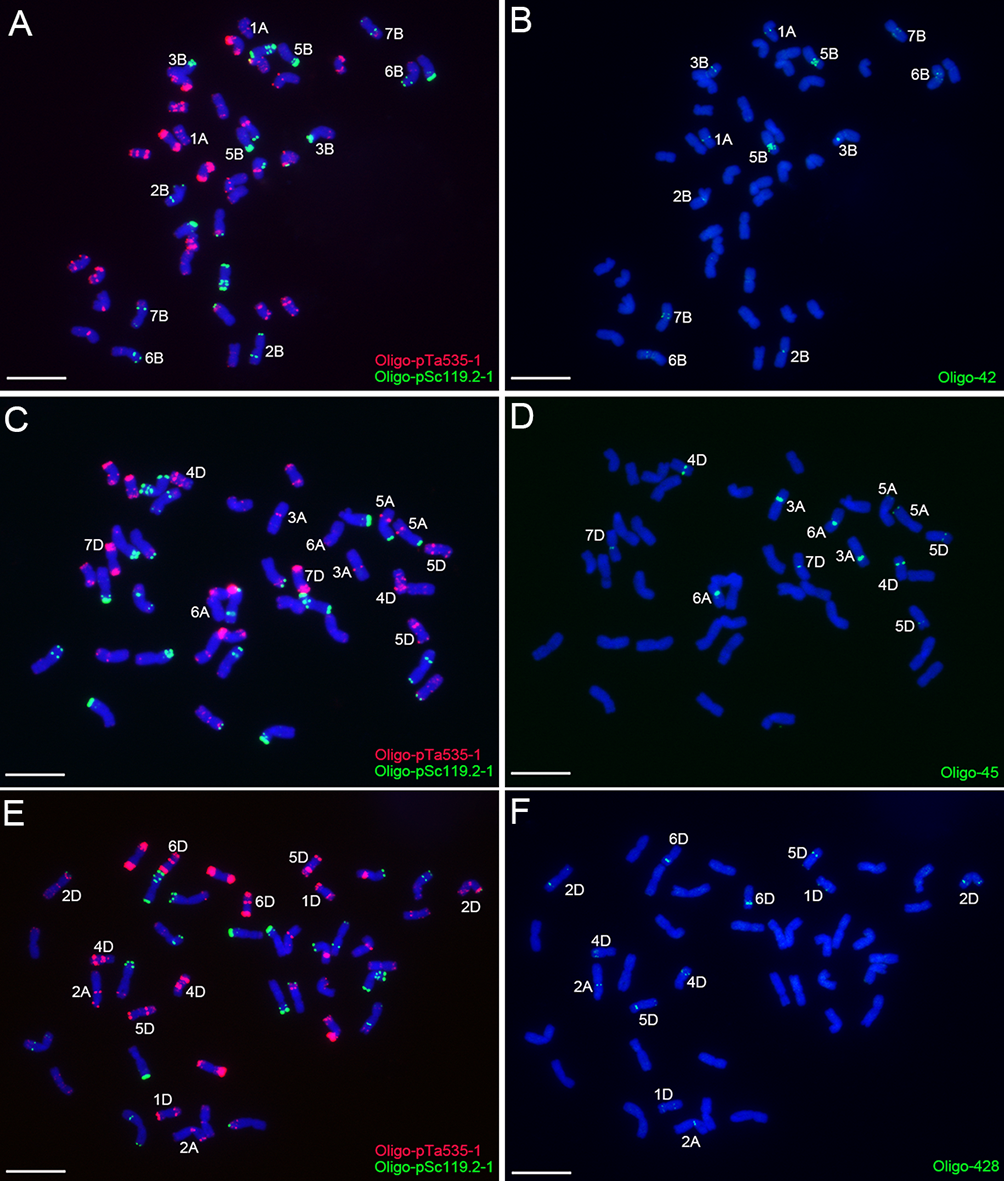

Supplement: FIGURE S12 — ND-FISH analysis of root tip metaphase chromosomes of wheat Chinese Spring. (A,B) ND-FISH analysis of the same cell using Oligo-pTa535-1 (red), Oligo-pSc119.2-1 (green), Oligo-42 (green) as probes. (C,D) ND-FISH analysis of the same cell using Oligo-pTa535-1 (red), Oligo-pSc119.2-1 (green), Oligo-45 (green) as probes. (E,F) ND-FISH analysis of the same cell using Oligo-pTa535-1 (red), Oligo-pSc119.2-1 (green), Oligo-448 (green) as probes. Chromosomes were counterstained with DAPI (blue). Scale bar: 10 μm. [file Image_12.TIF]
